# Development and validation of BATUTA: a test to evaluate the musical perception of people with hearing impairment

**DOI:** 10.1590/2317-1782/20232022010en

**Published:** 2023-08-11

**Authors:** Pierangela Nota Simões, Cristiano Miranda de Araújo, Guilherme Romanelli, Débora Lüders

**Affiliations:** 1 Programa de Pós-graduação em Distúrbios da Comunicação, Universidade Tuiuti do Paraná - UTP - Curitiba (PR), Brasil.; 2 Centro de Música e Musicoterapia, Universidade Estadual do Paraná - UNESPAR - Curitiba (PR), Brasil.; 3 Programa de Pós-graduação em Música, Universidade Federal do Paraná - UFPR - Curitiba (PR), Brasil.

**Keywords:** Music, Perception, Hearing Impairment, Hearing Aids, Validation, Test

## Abstract

**Purpose:**

To describe the development and validation of a test, called BATUTA, that assesses the musical perception of people with hearing impairment that are hearing aid (HA) users. BATUTA is a computerized test with 35 subtests, divided into the rhythm, pitch, and timbre modules, and the participants must answer whether the sound samples and/or parts of the songs, presented in pairs, are the same or not.

**Methods:**

The BATUTA creation process consisted of four stages: test development, submission to the expert committee for content validation; pilot application with 51 normal hearing participants and retest to validate reliability. The process was based on several recommendations for the development and validation of musical assessment instruments. A deep investigation of the guidelines related to sound samples used, musical attributes evaluated, testing environment and the most appropriate response method was undertaken to ensure dependability.

**Results:**

The Content Validity Index (CVI) and expert agreement rates, when analyzed with the committee's recommendations, resulted in corrections and new audio recordings to ensure compliance to the test. The pilot test scores indicated internal consistency and the retest confirmed the reliability of BATUTA.

**Conclusion:**

The results demonstrated the viability of BATUTA to assess the musical perception of people with hearing impairment that are HA users.

## INTRODUCTION

Noteworthy efforts have been undertaken in recent decades to enhance the speech perception of individuals with hearing impairment who use auxiliary hearing devices, whether Hearing Aids (HA) or Cochlear Implants (CI)^(1-3)^. However, the same progress has not been observed in musical perception, which tends to be difficult compared to the perception of individuals with normal hearing.

The explanation for the low quality of musical perception by users of auxiliary hearing devices is rooted in the acoustic characteristics of music, which are hard to transduce and result in distortion of the final output^(1)^. Furthermore, the spectral and temporal differences observed between speech and music contribute to increasing the contrast in musical perception by users of these devices^(4)^.

The human ear is sensitive to variations in phase, duration, and frequency, which translate into the sensation of pitch, present in the melody and harmony of music; and in the characteristics of rhythm related to the rate of repetition of sounds, as well as timbre, designated as the most complex of the musical elements because it integrates all others^(5)^.

The combination of the spectral elements pitch, melody, and harmony, and the temporal elements, along with timbre, makes music the most challenging of auditory stimuli^(4,6)^. Conversely, the richness of musical elements and the complexity of music convert the musical experience into a universal manifestation and highlight it as an important part of people's lives, whether they are hearing or not.

Therefore, music is a form of human expression with the potential to evoke memories and emotions. It is a facilitator for people to enjoy common interests and engage in collective activities. Furthermore, through music, people can interpret and assign meanings to their experiences and understand them better, which is why the effects of perception and appreciation of music have been pointed out as relevant to well-being^(7)^.

Given this perspective, several studies have been developed to understand the musical perception of people with hearing impairment, with particular emphasis on research focused on CI users^(6,8)^, and to evaluate this population's musical perception^(9-14)^.

Despite the gradual increase in access to CIs for the Brazilian population with hearing impairment, there is a significant predominance in the recommendation of HAs^(15)^. This situation justifies the development of research aimed at satisfying the users of this type of hearing device, including regarding the musical perception of this population.

Thus, this study aims to describe the development and validation process of a musical perception test called BATUTA^^1^^ , designed to evaluate the musical perception of people with hearing impairment who are HA users.

## METHODS

The development and validation of BATUTA followed recommendations for content validation during the instrument-building process^(16,17)^ and the guide for developing and validating tests in Speech Therapy^(18)^.

The process comprised four stages: (1) Test development; (2) Content validation by an expert committee; (3) Pilot test administration to participants with normal hearing to assess internal consistency; (4) Retest administration to validate reliability.

### Musical perception test development

BATUTA is a computerized test with 35 subtests categorized into rhythm, pitch, and timbre modules (Chart 1). Participants must indicate whether the sound samples or musical excerpts presented on the computer are the same or different^^2^^ when presented in pairs.

**Chart 1:** Legend of sound samples and song snippets from the BATUTA modules and subtests

**MODULE**	**SUBTEST**	**DESCRIPTION OF SOUND SAMPLES**
**RHYTHM**	Pulse	60bpm X 60bpm
		60bpm X 90bpm
		120bpm X 60bpm
	Tempo	60bpm X *acelerando*
		*ritardando* X 60
		*accelerando* X *accelerando*
	Meter	4/4 X3/4
		3/4 X 5/4
		3/4 X 3/4
**PITCH**	Melody	Asa Branca bassoon do 128 Hz X bassoon do 512 Hz
		Asa Branca flute la 880 Hz X flute la 880 Hz
		Asa Branca piano do 256 Hz X piano la 440 Hz
		Asa Branca violin la 220 Hz X violin la 440 Hz
		Asa Branca guitar do 128 Hz X guitar do 128 Hz
		Asa Branca piano la 440 Hz X piano la 880 Hz
		Asa Branca clarinet la 880 Hz X clarinet la 440Hz
		Asa Branca piano la 440 Hz X piano la 440 Hz
		Asa Branca cello do 256 Hz X la cello 440 Hz
		Asa Branca clarinet do 256 H X clarinet la 1760 Hz
	Harmony	Chords doM (256 Hz) X dom
		Chords doM (256 Hz) X doM
		Chords doM7m X laM7m (880 Hz)
		Chords laM7m (440 Hz) X laM7m (440 Hz)
		Chords doM x diminuto
		Chords laM7m (880 Hz) X laM7m (440 Hz)
**TIMBRE**		Ciranda cello X cello
Dó 256 Hz/C3		Ciranda piano X clarinet
		Ciranda violin X cello
		Ciranda piano X piano
		Ciranda cello X bassoon
		Ciranda piano X guitar
		Ciranda violin X violin
		Ciranda bassoon X violin
		Ciranda cello X piano
		Ciranda clarinet X clarinet

The BATUTA's development was preceded by a systematic review that uncovered the panorama of musical perception evaluation in people with hearing impairment^(19)^. Researchers from the Music and Audiology areas participated. These conditions are relevant procedures to ensure evidence of validity based on the test content^(18)^.

The systematic review enables the control of heterogeneity, a common issue in music research. It demonstrated that employing synthesizers to evaluate instrument recognition and melody can yield inaccurate outcomes. The reason for these inaccuracies is that synthesizers fall short of replicating the instruments’ authentic tonal quality, or 'timbre'^(19)^.

Therefore, the sound sample recordings of the pitch and timbre modules comprised real instruments played by professional musicians. They were converted into MP3 files.

The MP3 audio file recordings were converted to the MP4 audio and video standard, which were the basis for creating the videos generated in the Microsoft Photos video editor application. Then, the videos were uploaded to the YouTube video-sharing platform. Finally, their upload was made to the Google Forms research management application, where the first version of BATUTA was constructed.

**Figure 1:**
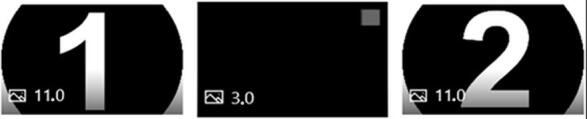
Storyboard of the sample presentation mode

The videos, whose duration ranges from 13 s to 28 s, present the first sound with the number 1, a brief pause with the black screen, and the second sound with the number 2 (Figure 1). After watching the video with no visual stimuli other than the numbers mentioned above, the participant must choose among the alternatives presented whether the sound is the same or different.

The sound samples of the rhythm module were generated in the Audacity® 2.3.1 software, with a xylophone timbre built using a sampler at the frequency of 1,000 Hz. Each sample has an average duration of 10 s, with small, allowed variations to preserve the sequences of stimuli and complete rhythmic cycles.

The rhythm module, designed to evaluate the element responsible for the speed and marking of beats and pauses in musical pieces, comprises pulse, tempo, and meter. The standard pulse for the subtest was set at 60 bpm beats, taken as a base one pulse per second, with subdivisions of 90 bpm and 120 bpm.

From the protocol established by the 60 s of constant pulse, the sound samples of the tempo subtest were calculated proportionally, with an increase of 160% for accelerando^^3^^ and a decrease of 55% for ritardando. In turn, the sample meter subtests record the beats and pauses in the 3/4, 4/4, and 5/4 formats.

Regarding the pitch module, the melody subtest was expressed by the first bars of the song Asa Branca^^4^^ played on the following instruments: cello, guitar, violin, piano, bassoon, flute, and clarinet, in the keys of C major and A major. In turn, the harmony subtest comprised recordings of the chords^^5^^ major, minor, and diminished, taking the notes C and A as fundamentals and playing on the piano.

It is worth mentioning the close relationship between melody and harmony with pitch, with the melody defined as the sequence of several pitches that comprise the musical phrase. Meanwhile, harmony comprises the vertical relationship between pitches, which, when played simultaneously, form the musical chords^(4)^.

The timbre module refers to the quality of sound and the discrimination of instruments playing the same musical notes. It was developed with recordings of the first bars of *Ciranda Cirandinha* ^^6^^ , played on the cello, guitar, violin, piano, bassoon, flute, and clarinet in the key of C3 (256 Hz).

### Content validation by the expert committee

Fourteen professional musicians, masters, and doctors in Music, or professionals of exceptional knowledge in the area, were invited to the committee of experts responsible for assessing the BATUTA's ability to accurately measure the phenomenon of musical perception^(16)^.

The invitation to the experts was made through an electronic message presenting BATUTA. After the musicians' positive response, they were sent the access link to BATUTA on Google Forms. The message exchange was private, and the experts, who had an average of 15 days to return the evaluation, worked individually and independently.

The committee's analysis comprised listening to the samples of each subtest and responding to the following questions through a Likert scale: (1) Stimulus presentation time: (1) adequate, (2) long, and (3) short; (2) Quality of the stimulus recording: (1) good, (2) regular, and (3) bad; (3) Fulfillment of the objective to which the test proposes: (1) fully fulfills, (2) partially fulfills, and (3) does not fulfill.

The experts had to answer five questions related to the test format: (1) The instructions for participants are; (2) The interface of BATUTA is; (3) The response format of BATUTA (equal/different) is; (4) The choice of songs is; (5) The total time required to answer BATUTA is. Response alternatives through a Likert scale: inadequate; somewhat adequate; reasonably adequate; and totally adequate.

The experts' involvement ended with an open-ended question: “What improvements are needed in BATUTA?”. This question was included in the evaluation process to allow them to offer suggestions and provide constructive feedback to enhance the test.

The Content Validation Index (CVI) was used to measure the percentage of judges who agreed on certain aspects of the instrument and its items. In cases where the CVI was less than the recommended value of 80%, the samples were excluded or reformulated^(17)^.

Furthermore, the CVI data were cross-referenced with the responses to the open-ended question using a methodological triangulation approach. It involved analyzing numerical indicators alongside the arguments put forth by the expert committee members^(16)^.

#### Pilot test application

The sample included students, teachers, employees of a school-clinic, patient companions, and family members. They agreed to participate in the research by signing the Free and Informed Consent Term.

Volunteers underwent audiometric evaluation before the BATUTA application. This evaluation involved conventional threshold tonal audiometry, assessing airway thresholds for frequencies ranging from 250 Hz to 8,000 Hz, and bone conduction for frequencies ranging from 500 Hz to 4,000 Hz in cases where airway thresholds exceeded 25 dB HL.

Inclusion criteria comprised: (1) having auditory thresholds up to 25 dB HL bilaterally in the researched frequencies, (2) being at least 18 years old, and (3) not having cognitive impairments that hindered discrimination of the “equal/different” concepts, as assessed during the test familiarization section. There were no gender or education level distinctions among the volunteers. The exclusion criteria were: (1) being an amateur or professional musician, (2) having previous music education, and (3) being a HA user.

The convenience sample comprised 51 volunteers who met the inclusion criteria^(17)^. Among the participants, 70.6% were female, and 29.4% were male. The sample’s age ranged from 19 to 55 years, with an average of 32.31 ± 10.82.

The pilot test application of BATUTA was carried out in a quiet room^(21)^, with stimulus presentation through a speaker positioned at 0º Azimuth^(14)^ and 1 m away from the participant^(9,22,23)^, at an intensity of 70 dBA as measured by a decibel meter^(21,22)^. The computer used for testing was a Lenovo Yoga 520-14IKB notebook, combined with a 30-watt RMS Bose SoundTouch 10 wireless speaker.

Participants underwent a familiarization session^(23)^ before the testing, which the same administrator supervised throughout the process.

The responses were tabulated in Microsoft Excel (version 16.0). A value of 1 (one) was assigned to correct responses, and a value of 0 (zero) was assigned to incorrect responses. The total number of correct answers for the 35 sound samples and for the rhythm, pitch, and timbre modules was calculated using inferential statistics.

Given that BATUTA is an instrument with dichotomous responses (same/different), despite the Cronbach's alpha coefficient being the most well-known measure in evaluating internal consistency, the Kuder-Richardson (KR-20) test was applied, which is used as a reference for evaluating the internal consistency of instruments that use this type of variables^(24)^.

#### Reliability validation

The BATUTA's consistency in producing consistent results over time and space was evaluated through a retest. Fourteen participants, randomly selected from the initial sample, were invited to complete the same version of BATUTA.

The retest was conducted approximately 20 days after the initial test, which was deemed sufficient to prevent test recall and ensure no clinical changes had occurred in the participants^(18)^. The agreement of participant responses at the two different times was assessed using the Kappa coefficient (k).

## RESULTS

### Content validation

The experts’ responses were analyzed for the Content Validity Index (CVI) and the percentage of agreement among the committee members^(16,17)^. The CVI scores for the BATUTA modules were 80% for rhythm, 75% for pitch, and 86% for timbre. Table 1 presents the evaluation results for each BATUTA subtest.

**Table 1:** Questionnaire results with expert responses and CVI index (n=14)

MODULE	SUBTEST	QUESTION	RESPONSE FREQUENCIES			CVI
			Item 1	Item 2	Item 3	
Rhythm	Pulse	Timing	12	2	-	86%
		Quality	11	3	-	79%
		Objective	14	-	-	100%
	Tempo	Timing	14	-	-	100%
		Quality	11	3	-	79%
		Objective	11	2	1	79%
	Meter	Timing	11	-	3	79%
		Quality	12	2	-	86%
		Objective	9	3	2	64%
*Pitch*	Harmony	Timing	14	-	-	100%
		Quality	7	4	3	50%
		Objective	10	3	1	71%
	Melody	Timing	12	2	-	86%
		Quality	11	3	-	79%
		Objective	13	1	-	93%
Timbre	Timbre	Timing	12	2	-	86%
		Quality	11	3	-	79%
		Objective	13	1	-	93%

Despite the high CVI score for the rhythm module, the experts pointed out problems in meeting the objectives of the meter subtest (64%).

The CVI data for the recording quality analysis and objectives of the harmony subtest recorded scores below 78%, resulting in poor performance for the pitch module (75%).

Table 2 presents the percentage of agreement among the expert committee members for the analysis of aspects related to the test format.

**Table 2:** Positive agreement (%) regarding the overall test format (n=14)

QUESTION	RESPONSES				%
	Totally adequate	Reasonably adequate	Somewhat adequate	Inadequate	
Instruçtions	10	3	1	-	93%
Interface	11	4	-	-	100%
Response format	10	5	-	-	100%
Choice of song	12	2	-	-	100%
Time required	9	6	-	-	100%

Once the quantitative phase of BATUTA's content validation was completed, the qualitative analysis of the descriptive responses provided by the expert committee on “What could be improved in BATUTA” began. Thus, the observations and recommendations of the experts were carefully read and analyzed, as described in Chart 2, to list categories constituted from their comments^(25)^.

**Chart 2:** Categorization and excerpts from the speeches of experts and the adjustments promoted

CATEGORIES	EXTRACTS THAT OBSERVE AND RECOMMEND	ADJUSTMENTS
Duration of samples	(O) long samples for the intended objective/extended recording duration	Formatting of musical phrases
	(R) longer pause time between samples	Formatting of videos with adjustment of the presentation time of recordings and pauses
	(O) tempo difference between recordings	Control of recording tempo using *Audacity* ® 2.3.1 software
Audio quality	(O) noise in the samples	Application of the noise reduction effect by the Audacity ® 2.3.1 software
	(O) echo	Application of fade in and fade out features
	(O) hiss in the background	
	(O) abrupt cut off of sounds	
Intensity of samples	(O) recordings with louder volume than the others	Normalization of recordings with the MP3Gain 1.3.4 software
	(O) timbre test samples with different intensities	
	(O) difference between the 'volumes' of some recordings	

Therefore, the recording of the sound stimuli of the harmony subtest, whose quality was classified as regular by four experts and poor by one of them, was corrected with new recordings. Regarding the compliance of this same subtest, it was possible to relate the results of the experts' evaluation to the quality of the audio files based on comments about the presence of echo in the samples. The re-recording corrected the problems pointed out for the harmony item.

The experts' observations regarding the instructions and the guidelines at the beginning of the test or before the presentation of the sound samples resulted in more detailed instructions at the beginning of each module and/or subtest.

### Pilot test application

After the adjustments, the pilot test was applied with the 51 participants, who listened to each of the 35 sound samples and answered “same/different” to the questions regarding the modules: (1) rhythm: Are the samples?; (2) pitch: Are the song snippets?/Are the chords?; (3) timbre: Are song snippets played by instruments?

The participant and the examiner were in the room during the pilot test^^7^^ . The average response time was 20 minutes, and repetitions were allowed, although not encouraged.

### Pilot test results

Table 3 describes the results of the 51 participants, considering the values of 1 (one) and 0 (zero) assigned for the “same/different” responses of the 35 sound samples and the BATUTA modules.

**Table 3:** Mean, standard deviation, median, minimum, and maximum values of evaluated samples (n=51)

	n	mean	standard deviation	median	minimum	maximum
Rhythm	9	8.4313	0.8307	9	7	9
Pitch	16	15.0980	1.2042	16	11	16
Timbre	10	9.8235	0.4338	10	08	10
Total	35	33.3594	3.5355	34	29	35

Regarding participants’ performance in the test, the results revealed that the lowest index was 82%, corresponding to the correct response for 29 samples, and 54.89% of the participants obtained results above average. Table 4 shows the number of correct answers, the correct answer index, and the proportion of participants with equal or higher values for each range of correct answers.

**Table 4:** Results of the BATUTA pilot test (n=51)

Number of correct answers by participants	Percentage of correct answers by participants	Number of participants who answered correctly	Percentage of participants who answered correctly	Percentage of participants who answered with an equal or higher number of correct answers.
29	82%	2	3.92%	100%
30	85%	3	5.88%	96.05%
31	88%	4	7.84%	90.17%
32	91%	5	9.80%	82.33%
33	94%	9	17.64%	72.53%
34	97%	8*	15.68%*	54.89%*
35	100%	20*	39.21%*	39.21%*

* Percentage of participants who presented results above the average

### Internal consistency validation

The Kuder-Richardson test (KR-20) was used in evaluating the internal consistency of the pilot test. The result for the 35 questions with dichotomous responses, expressed as “same/different”, as estimated by statistical analysis, was 0.62.

### Reliability validation

The reliability of BATUTA was validated through a retest test conducted with 14 participants drawn from the initial group. The results of the two applications of the test were used to calculate the Kappa coefficient (K), which resulted in a value of 0.89.

## DISCUSSION

Despite the availability of many assessment tools for speech therapy, only a few undergo the validation process to gather evidence for their endorsement^(18)^. Furthermore, there is a lack of guidelines for constructing and using tests in Speech Therapy^(26)^.

Regarding musical perception, the national literature includes an instrument designed to assess the recognition of traditional Brazilian melodies and examine the performance of children with normal hearing^(27)^.

BATUTA, in this same trend, presents the uniqueness of containing excerpts from the Brazilian folk songbook and is the first musical perception test that evaluates the attributes of rhythm, pitch, and timbre developed for the Brazilian population. Since music is not a culturally neutral phenomenon, it is reasonable to consider this a promising aspect of the test.

The performance of a systematic review on musical perception tests in people with hearing impairment before the construction of BATUTA produced evidence that allowed us to overcome difficulties encountered in previous studies related to the heterogeneity in music^(6)^. Moreover, it was possible to systematize guidelines regarding musical elements evaluated, test environment, mode of presentation of sound stimuli, and type of response suitable for the proposed testing to structure the concepts and the argument of the function measured for the elaboration of a robust construct^(19)^.

An example illustrating this is the result of a meta-analysis, which revealed that cochlear implant (CI) users face greater difficulty perceiving melody compared to timbre, particularly when timbre is assessed using digitized sounds and melody tests are conducted with synthesized samples^(19)^. Although the study focused on CI users, these findings can be applied to the type of sound stimuli used in the tests. The filters and algorithms employed in HA and CI programming restrict the dynamic range, making it more challenging to perceive synthesized sounds through HAs. Consequently, this finding motivated the decision to record sound samples for the pitch and timbre modules using real instruments instead of generating synthesized sounds.

Since content validation plays a crucial role in the selection and application of an instrument, the experts chosen for this stage were selected based on their training, qualifications, and availability. These professionals were considered experts in the field and acted as judges, evaluating and confirming the clarity, relevance, and fidelity of BATUTA^(24)^.

The percentage of agreement among the committee of experts regarding the format of the test was above 90% for all questions, which is desirable^(16)^. Since there was no response for inadequate, it can be concluded that the experts' interaction with BATUTA was good.

The triangulation of the CVI results with the categories, or thematic axes, proposed from the responses of what could improve in BATUTA allowed the correlation of objective data with descriptive content. Furthermore, it ensured rigor and objectivity in the analysis of the arguments expressed by the experts. From these data, it was possible to implement improvements in sound samples to achieve the proposed objectives.

Results show distinct hearing patterns among laypersons, students, and music teachers. Notably, teachers demonstrated a wider range of technical criteria for performance analysis^(28)^. It suggests that the experts were meticulous in evaluating BATUTA, and adherence to their recommendations indicates test quality.

The analysis of the pilot test results showed that 54.89% of the participants scored above average for the 35 items surveyed and that even those with lower results obtained a fair number of correct answers. In other words, participants who answered 29 questions correctly had an accuracy rate of 82%.

Based on this context, it can be concluded that the participants' responses were consistent. The BATUTA's basis on protocols designed to assess musical perception in individuals with hearing impairment, coupled with the consistent data obtained during the pilot test, indicates the feasibility of using BATUTA to evaluate the musical perception of individuals with HI who are HA users.

The testing conditions recommended for individuals with hearing impairment who use HA are similar to those described in the methodology applied to normal hearing participants, except for stimulus intensity. Studies included in the systematic review propose that HI participants should adjust the volume of the stimuli presented through a speaker to a comfortable audibility level^(19)^.

The reliability of BATUTA was assessed by examining the consistency of measurements under test-retest conditions. The agreement of answers among participants was evaluated using the Kappa coefficient, which ranges from -1 to 1^(29)^. An interpretation of the coefficient suggests that a value of 0.89 indicates excellent agreement and represents the reliability of BATUTA^(24)^.

The Kappa coefficient was chosen due to its recommendation for evaluating agreement measures in the healthcare field, particularly in instruments with nominal categories^(16)^.

Reliability is referred to by several terms, such as fidelity, equivalence, consistency, objectivity, reproducibility, stability, and homogeneity, depending on the literature and the aspect of the test being emphasized^(30)^.

For assessing internal consistency, options include the Kuder-Richardson test and Cronbach's alpha coefficient. While the alpha coefficient is commonly used, the Kuder-Richardson (KR-20) technique is recommended for scales with dichotomous responses like BATUTA, which uses the options of “same/different”.

Regarding interpretation, both Cronbach's alpha coefficient and Kuder-Richardson values above 0.70 are considered ideal, although this value is not universally accepted. Some studies suggest that values close to 0.60 are satisfactory, leading to the acceptance of BATUTA's internal consistency with a result of 0.62^(17)^.

In summary, the interpretation of BATUTA results proposes that each correct answer is awarded 1.0 point, and the final scores are analyzed as follows: ≥ 33 correct (above 94%): excellent musical perception; 29 to 32 correct: good musical perception; 25 to 28 correct: reasonable musical perception; ≤ 24 correct (below 68%): difficulty in musical perception.

## CONCLUSION

The development of BATUTA was presented, including the theory and construct behind it, the reasons for its creation, and the intended target population.

Adherence to established guidelines in tests and protocols for assessing musical perception in individuals with hearing impairment, along with the results of content validation, internal consistency, and reliability stages of the pilot test conducted with individuals with normal hearing, indicated the suitability of BATUTA for evaluating musical perception in individuals with HI who use HA.

BATUTA is suitable for use in its intended population. Future studies can compare musical perception between individuals with normal hearing and those with hearing impairment, between hearing aid users with specific adjustments for music appreciation, and between hearing aid users and cochlear implant users. Furthermore, they can explore other possibilities related to researching musical perception in this population.

BATUTA has the potential to offer an innovative perspective in speech therapy, both in the selection and recommendation of HA and in monitoring users of assistive hearing devices who seek to engage with the musical realm.

## Footnotes

1 From Italian battuta. Feminine noun: short wand with which conductors lead orchestras. Adjective: Comrade; reliable. Noun: Something excessively good; exquisite.

2 The Forced choice method is a response pattern used to measure subjective experiences^(20)^.

3 Italian words are commonly used in musical notation to indicate to performers how the music should be played; Italian composers first used them in the 17th century and have spread worldwide ever since. The terms accelerando and ritardando are related to tempo changes and mean “gradually faster” and “gradually slower” in that order.

4 Luiz Gonzaga and Humberto Teixeira, 1947.

5 Chords are groups of notes played simultaneously and combined according to an order and logic that define the harmony of the music. The structure of a chord comprises three musical notes: the root note, or tonic, a third, and a fifth. There are different types of thirds and fifths, and their combinations can generate many chords, such as Major, Minor, and *Diminuto*.

6 A Brazilian folk song.

7 The sanitary and educational protocols for COVID-19 prevention were fully complied with during the application.
