# Supplementary material for: Development and validation of BATUTA: a test to evaluate the musical perception of people with hearing impairment
Source: Codas. 2023 Aug 11;35(4):e20220010. doi: 10.1590/2317-1782/20232022010en (PMC10446752; doi:10.1590/2317-1782/20232022010en)
Supplement: Translation

# Translation: Desenvolvimento e validação do BATUTA: um teste para avaliação da percepção musical de pessoas com deficiência auditiva

**DOI:** 10.1590/2317-1782/20232022010pt

**Authors:** Pierangela Nota Simões, Cristiano Miranda de Araújo, Guilherme Romanelli, Débora Lüders

**Affiliations:** 1 Programa de Pós-graduação em Distúrbios da Comunicação, Universidade Tuiuti do Paraná - UTP - Curitiba (PR), Brasil.; 2 Centro de Música e Musicoterapia, Universidade Estadual do Paraná - UNESPAR - Curitiba (PR), Brasil.; 3 Programa de Pós-graduação em Música, Universidade Federal do Paraná - UFPR - Curitiba (PR), Brasil.

**Keywords:** Música, Percepção, Deficiência Auditiva, Aparelho Auditivo, Validação, Teste

## Abstract

**Objetivo:**

Descrever o desenvolvimento e a validação de um teste de percepção musical, denominado BATUTA, destinado a avaliar a percepção musical de pessoas com deficiência auditiva, usuárias de aparelhos de amplificação sonora individual (AASI). O BATUTA é um teste computadorizado composto por 35 subtestes divididos nos módulos ritmo, *pitch* e timbre para os quais os participantes devem responder se as amostras sonoras e/ou os trechos musicais apresentados, aos pares, são iguais ou diferentes.

**Método:**

O processo de construção do BATUTA foi composto por quatro etapas: desenvolvimento do teste, submissão da versão inicial ao comitê de especialistas para validação de conteúdo; aplicação do piloto em 51 participantes com audição normal e reteste para validação da confiabilidade, fundamentadas em reconhecidas recomendações para elaboração e validação de instrumentos de avaliação. Diretrizes relacionadas à natureza das amostras sonoras utilizadas, aos atributos musicais avaliados, ao ambiente de testagem e ao tipo de resposta indicada para a finalidade do BATUTA foram amplamente investigadas, com o propósito de lhe conferir fidedignidade.

**Resultados:**

Os índices de Validação de Conteúdo (IVC) e de concordância entre os especialistas, quando analisados juntamente com as recomendações do comitê, resultaram em correções e em novas gravações dos áudios para garantir o cumprimento do teste. Os escores da aplicação do teste piloto indicaram boa consistência interna e o reteste confirmou a confiabilidade do BATUTA.

**Conclusão:**

Os resultados demonstraram a viabilidade do BATUTA para avaliar a percepção musical de pessoas com deficiência auditiva usuárias de AASI.

## INTRODUÇÃO

Notáveis esforços foram empreendidos nas últimas décadas para melhorar a percepção da fala de pessoas com deficiência auditiva, usuárias de dispositivos auditivos auxiliares, sejam eles aparelhos de amplificação sonora individual (AASI) ou implantes cocleares (IC)^(1-3)^. Contudo, o mesmo progresso não foi observado no domínio da percepção musical, que tende a ser difícil quando comparada à percepção de indivíduos ouvintes.

A explicação para a baixa qualidade na percepção musical por usuários de dispositivos auditivos auxiliares está fundamentada em características acústicas da música, que são de difícil transdução e resultam na distorção do resultado final^(1)^. Adicionalmente, as diferenças espectrais e temporais observadas entre a fala e a música contribuem para acentuar o contraste na percepção musical por usuários desses dispositivos^(4)^.

O ouvido humano é sensível a variações sonoras de fase, duração e frequência, que se traduzem em termos da sensação de tonalidade *(pitch*), presente na melodia e na harmonia da música; e nas características do ritmo, relacionado à taxa de repetição dos sons, tanto quanto do timbre, designado como o mais complexo dos elementos musicais por integrar todos demais^(5)^.

O conjunto dos elementos espectrais *pitch*, melodia e harmonia e dos elementos temporais, somados ao timbre, faz da música o mais desafiador dos estímulos auditivos^(4,6)^. Em contrapartida, a riqueza dos elementos musicais e a complexidade da música, convertem a experiência musical em uma manifestação universal e a evidenciam como uma parte importante da vida das pessoas, sejam elas ouvintes ou não.

Destaca-se, assim, o papel da música como uma relevante forma de expressão humana, com potencial para evocar lembranças e emoções, além de funcionar como um facilitador para que as pessoas desfrutem de interesses em comum e realizem atividades coletivamente. Ademais, por meio da música as pessoas conseguem interpretar e atribuir significados para suas experiências e compreendê-las melhor, razão pela qual os efeitos da percepção e a apreciação musical têm sido apontadas como relevantes para o bem-estar^(7)^.

Diante dessa perspectiva, vários estudos foram desenvolvidos com a finalidade de compreender a percepção musical de pessoas com deficiência auditiva, sendo dado maior destaque para pesquisas focadas em usuários de IC^(6,8)^, assim como para avaliar a percepção musical desse público^(9-14)^.

Apesar do crescimento gradual no acesso da população brasileira com deficiência auditiva ao IC, há uma significativa predominância na indicação de AASI^(15)^, circunstância que justifica o desenvolvimento de pesquisas voltadas à satisfação dos usuários de desse tipo de dispositivo auditivo, inclusive no que se refere à percepção musical desta população.

Assim, o presente estudo tem como objetivo descrever o processo de desenvolvimento e de validação de um teste de percepção musical, denominado BATUTA^^1^^ , destinado a avaliar a percepção musical de pessoas com deficiência auditiva, usuárias de AASI.

## MÉTODO

O desenvolvimento e a validação do BATUTA foram baseados nas recomendações para validação de conteúdo durante os processos de construção de instrumentos^(16,17)^ e no guia de recomendações para elaboração e processo de validação de testes em Fonoaudiologia^(18)^.

O processo se deu em quatro etapas: (1) Desenvolvimento do teste (2); Submissão do teste ao comitê de especialistas para validação de conteúdo; (3) Aplicação do teste piloto em participantes com audição normal para validação de coerência interna; (4) Aplicação do reteste para validação de confiabilidade.

### Desenvolvimento do teste de percepção musical BATUTA

O BATUTA é um teste computadorizado composto por 35 subtestes divididos nos módulos ritmo, *pitch* e timbre (Quadro 1), para os quais os participantes devem responder se as amostras sonoras e/ou os trechos musicais apresentados no computador, aos pares, são iguais ou diferentes^^2^^ .

**Quadro 1:** Legenda das amostras sonoras e trechos de canções dos módulos e subtestes do BATUTA

**MÓDULO**	**SUBTESTE**	**DESCRIÇÃO DA AMOSTRA SONORA**
**RITMO**	Pulso	60bpm X 60bpm
		60bpm X 90bpm
		120bpm X 60bpm
	Andamento	60bpm X *acelerand*o
		*ritardando* X 60
		*accelerando* X *accelerando*
	Compasso	4/4 X3/4
		3/4 X 5/4
		3/4 X 3/4
***PITCH***	Melodia	Asa Branca fagote dó 128 Hz X fagote dó 512 Hz
		Asa Branca flauta lá 880 Hz X flauta lá 880 Hz
		Asa Branca piano dó 256 Hz X piano lá 440 Hz
		Asa Branca violino lá 220 Hz X violino lá 440 Hz
		Asa Branca violão dó 128 Hz X violão dó 128 Hz
		Asa Branca piano lá 440 Hz X piano lá 880 Hz
		Asa Branca clarinete lá 880 Hz X clarinete lá 440Hz
		Asa Branca piano lá 440 Hz X piano lá 440 Hz
		Asa Branca violoncelo dó 256 Hz X violoncelo lá 440 Hz
		Asa Branca clarinete dó 256 H X clarinete lá 1760 Hz
	Harmonia	Acordes dóM (256 Hz) X dóm
		Acordes dóM (256 Hz) X dóM
		Acordes dóM7m X láM7m (880 Hz)
		Acordes láM7m (440 Hz) X láM7m (440 Hz)
		Acordes dóM x diminuto
		Acordes láM7m (880 Hz) X láM7m (440 Hz)
**TIMBRE**		Ciranda violoncelo X violoncelo
Dó 256 Hz		Ciranda piano X clarinete
		Ciranda violino X violoncelo
		Ciranda piano X piano
		Ciranda violoncelo X fagote
		Ciranda piano X violão
		Ciranda violino X violino
		Ciranda fagote X violino
		Ciranda violoncelo X piano
		Ciranda clarinete X clarinete

O desenvolvimento do BATUTA foi precedido por uma revisão sistemática que desvendou o panorama da avaliação da percepção musical em pessoas com deficiência auditiva^(19)^ e contou com a participação de pesquisadores das áreas da Música e da Audiologia, condições apontadas como procedimentos de relevância para garantir evidências de validade baseada no conteúdo do teste^(18)^.

Tal estudo possibilitou o controle da heterogeneidade presente em estudos que envolvem a música e evidenciou que o uso de sintetizadores para avaliar o reconhecimento de instrumentos e a melodia pode levar a resultados equivocados, uma vez que não são capazes de reproduzir o timbre original dos instrumentos^(19)^.

Portanto, as gravações das amostras sonoras dos módulos de *pitch* e timbre foram realizadas com instrumentos reais, tocados por músicos profissionais e, posteriormente, convertidas em arquivos MP3.

As gravações em arquivos de áudio no formato MP3 foram convertidas para o padrão de áudio e vídeo MP4, que foram a base para criação dos vídeos gerados no editor de vídeo do aplicativo *Microsoft Photos*. Em seguida, os vídeos foram enviados para a plataforma de compartilhamento de vídeo YouTube e, por último, foi realizado seu *upload* para o aplicativo de gerenciamento de pesquisas *Google Forms*, onde foi construída a primeira versão do BATUTA.

Os vídeos, cuja duração varia de 13s a 28s, apresentam o primeiro som acompanhado do número 1, uma pausa breve acompanhada da tela preta, e o segundo som acompanhado do número 2 (Figura 1). Após assistir o vídeo, que não contém estímulos visuais além dos números já mencionados, o participante deve escolher dentre as alternativas apresentadas, se o som é igual ou diferente.

**Figura 1:** *Storyboard* do modo de apresentação das amostras

As amostras sonoras do módulo ritmo foram geradas no programa *Audacity*® 2.3.1, com timbre de xilofone construído por meio de *sampler* na frequência de 1.000 Hz. Cada amostra tem o tempo médio de 10s, com pequenas variações consentidas para preservar as sequências dos estímulos e ciclos rítmicos completos.

O módulo ritmo, destinado a avaliar o elemento responsável pela velocidade e pela marcação das batidas e das pausas nas peças musicais, consiste nos subtestes pulso, andamento e compasso. O pulso padrão para o subteste foi convencionado em batidas de 60 bpm, tomado como base um pulso por segundo, com desdobramentos de 90 bpm e 120 bpm.

A partir do protocolo estabelecido pelos 60s do pulso constante, as amostras sonoras do subteste andamento foram calculadas, proporcionalmente, com aumento de 160% para *accelerando* ^^3^^ e diminuição de 55% para *ritardando*. As amostras do subtestes compasso, por sua vez, registram as batidas e as pausas nos formatos 3/4, 4/4 e 5/4.

No que se refere ao módulo *pitch*, o subteste melodia foi expresso pelos primeiros compassos da canção Asa Branca^^4^^ tocados nos seguintes instrumentos: violoncelo, violão, violino, piano, fagote, flauta e clarinete, nas tonalidades dó maior e lá maior. O subteste harmonia, por sua vez, consistiu em gravações dos acordes^^5^^ maior, menor, diminuto, tomando as notas dó e lá, enquanto fundamentais e executadas ao piano.

É interessante observar a estreita relação entre a melodia e a harmonia com o *pitch*, sendo a melodia definida como a sequência de vários *pitches* que formam a frase musical, ao passo que a harmonia consiste na relação vertical entre os *piches*, que tocados simultaneamente formam os acordes musicais^(4)^.

O módulo timbre, que se refere à qualidade do som e à discriminação de instrumentos que tocam as mesmas notas musicais, foi desenvolvido com gravações dos primeiros compassos de Ciranda Cirandinha^^6^^ , tocados ao violoncelo, violão, violino, piano, fagote, flauta e clarinete, na tonalidade dó 256 Hz.

### Submissão do teste ao comitê de especialistas para validação de conteúdo

Foram convidados 14 músicos profissionais, mestres e doutores em Música, ou profissionais de *notório saber* na área, para compor o comitê de especialistas responsável pela avaliação da capacidade do BATUTA em medir com precisão o fenômeno da percepção musical^(16)^.

O convite aos especialistas se deu por meio de uma mensagem eletrônica para apresentação do BATUTA e, posteriormente à resposta positiva dos músicos em participar, lhes foi encaminhado o *link* de acesso ao BATUTA no *Google Forms*. A troca de mensagens foi privada e os especialistas, que tiveram um prazo médio de 15 dias para retorno da avaliação, trabalharam de forma individual e independente.

A análise do comitê consistiu na audição das amostras de cada subteste e nas respostas das seguintes questões por meio de uma escala *Likert*: (1) Tempo de apresentação dos estímulos (1) adequado (2) longo (3) curto; (2) Qualidade da gravação dos estímulos (1) boa (2) regular (3) ruim (3); Cumprimento do objetivo a que o teste se propõe (1) cumpre totalmente (2) cumpre parcialmente (3) não cumpre.

Os especialistas também foram solicitados a responder cinco questões, relativas ao formato do teste: (1) *As instruções para os participantes são*; (2) *A interface do BATUTA é*; (3) *O formato de resposta do BATUTA (igual/diferente) é*; (4) *A escolha das canções é;* (5) *O tempo total necessário para responder o BATUTA é*. Com alternativas de resposta, por meio de escala *Likert*: inadequado; pouco adequado; razoavelmente adequado; e totalmente adequado.

A participação dos especialistas foi finalizada com uma questão aberta: *O que precisa melhorar no BATUTA?* A inserção desta pergunta na avaliação teve como objetivo lhes proporcionar um espaço para fazer sugestões e fornecer um *feedback* produtivo para o aperfeiçoamento do teste.

O Índice de Validação de Conteúdo (IVC) foi utilizado para medir a porcentagem de juízes que estava em concordância sobre determinados aspectos do instrumento e de seus itens. Nos casos em que o IVC foi inferior ao valor recomendado de 80%, as amostras foram excluídas ou reformuladas^(17)^.

Adicionalmente, os dados do IVC foram correlacionados com as respostas expressas na última pergunta do teste piloto, por meio da triangulação metodológica dos indicadores numéricos e da argumentação oferecida pelos membros do comitê de especialistas^(16)^.

### Aplicação do teste piloto em participantes com audição normal

A amostra foi composta por estudantes, docentes e funcionários de uma clínica-escola, além de acompanhantes de pacientes e familiares, que expressaram sua concordância em participar da pesquisa por meio da assinatura do Termo de Consentimento Livre e Esclarecido (TCLE), aprovado pelo comitê de ética conforme parecer consubstanciado n. 3.468.404.

Os voluntários foram submetidos à avaliação audiométrica previamente à aplicação do BATUTA, por meio da realização de audiometria tonal liminar convencional, com pesquisa dos limiares de via aérea para as frequências de 250 Hz a 8.000 Hz, e de via óssea para as frequências de 500 Hz a 4.000 Hz, nos casos em que os limiares de via aérea apresentaram valores superiores a 25 dB NA.

Como critérios de inclusão, foram estabelecidos (1) apresentar resultado dos limiares auditivos de até 25 dB NA nas frequências pesquisadas, bilateralmente; (2) ter idade mínima de 18 anos e (3) não apresentar alterações cognitivas que impedissem a discriminação dos conceitos igual/diferente, detectáveis na seção de familiarização com o teste. Ainda, não foram feitas distinções de gênero e grau de escolaridade entre os voluntários. Os critérios de exclusão adotados foram: (1) ser músico amador ou profissional; (2) ser ou ter sido estudante de música e (3) ser usuário de dispositivos auditivos auxiliares.

Cinquenta e um voluntários que cumpriram os critérios de inclusão compuseram uma amostra de conveniência^(17)^. Dentre os participantes, 70,6% eram do gênero feminino e 29,4% do gênero masculino. A idade da amostra variou entre 19 e 55 anos, com média de 32,31 ± 10,82.

A aplicação do teste piloto do BATUTA foi realizada em uma sala silenciosa^(21)^, com apresentação dos estímulos por meio de uma caixa acústica posicionada à 0º Azimute^(14)^ e a 1m de distância do participante^(9,22,23)^, na intensidade de 70 dBA aferida por decibelímetro^(21,22)^. O computador utilizado para a testagem foi o *notebook Lenovo Yoga 520-14IKB*, combinado à caixa acústica *Bose SoundTouch 10 wireless speaker de 30 wats RMS*.

Houve uma sessão de familiarização dos participantes previamente à testagem^(23)^, que foi acompanhada pela mesma aplicadora durante todo o processo.

As respostas foram tabuladas no *Microsoft Excel* (versão 16.0). Quando a resposta correta era *igual* foi atribuído o valor 1 (um) quando a resposta foi *igual* e 0 (zero) quando a resposta foi *diferente*, em toda a coluna correspondente à amostra sonora avaliada. No caso de a resposta correta ser *diferente*, foi atribuído o valor o 1 (um) quando a resposta foi *diferente* e 0 (zero) quando a resposta foi *igual.* A soma dos acertos para as 35 amostras sonoras, tanto quanto para os valores dos módulos ritmo, *pitch* e timbre, foram calculadas por meio de estatística inferencial.

Sendo o BATUTA um instrumento com respostas dicotômicas (igual/diferente), apesar do coeficiente de *alfa de Cronbach* ser a medida mais conhecida na avaliação da consistência interna, foi aplicado o teste *Kuder-Richardson* (KR-20), utilizado como referência para avaliar a consistência interna de instrumentos que contam com esse tipo de variáveis^(24)^.

### Realização do reteste com um grupo de participantes para validação da confiabilidade

A capacidade do BATUTA em reproduzir um resultado de forma consistente, no tempo e no espaço, foi mensurada por meio da aplicação do reteste. Foram convidados 14 participantes, escolhidos aleatoriamente dentre os que constituíram a amostra inicial, para responderem a mesma versão do BATUTA.

A nova administração do BATUTA se deu cerca de 20 dias após a primeira testagem, sendo este um período que foi considerado longo o suficiente para evitar a lembrança do teste e curto o suficiente para garantir que não tenha ocorrido nenhuma mudança clínica nos participantes^(18)^. O coeficiente de *Kappa* (k) foi calculado para medir a concordância das respostas dos participantes nos dois momentos distintos.

## RESULTADOS

### Validação de conteúdo

As respostas dos especialistas foram analisadas quanto ao IVC e à porcentagem de concordância entre os membros do comitê^(16,17)^. Os escores do IVC para os módulos BATUTA foram ritmo 80%, *pitch* 75% e timbre 86% e os resultados da avaliação para cada subteste do BATUTA estão relacionados na Tabela 1.

**Tabela 1:** Resultados do questionário com as respostas dos especialistas e o cálculo do IVC (n=14)

MÓDULO	SUBTESTE	QUESTÃO	FREQUÊNCIAS DAS RESPOSTAS			IVC
			Item 1	Item 2	Item 3	
Ritmo	Pulso	Tempo	12	2	-	86%
		Qualidade	11	3	-	79%
		Objetivos	14	-	-	100%
	Andamento	Tempo	14	-	-	100%
		Qualidade	11	3	-	79%
		Objetivos	11	2	1	79%
	Compasso	Tempo	11	-	3	79%
		Qualidade	12	2	-	86%
		Objetivos	9	3	2	64%
*Pitch*	Harmonia	Tempo	14	-	-	100%
		Qualidade	7	4	3	50%
		Objetivos	10	3	1	71%
	Melodia	Tempo	12	2	-	86%
		Qualidade	11	3	-	79%
		Objetivos	13	1	-	93%
Timbre	Timbre	Tempo	12	2	-	86%
		Qualidade	11	3	-	79%
		Objetivos	13	1	-	93%

Apesar do alto índice de IVC para o módulo de ritmo, os especialistas apontaram problemas no cumprimento dos objetivos do subteste compasso (64%).

Os dados do IVC, para a análise da qualidade de gravação e objetivos do subteste harmonia registraram escores abaixo de 78% e resultaram em baixo desempenho para o módulo de *pitch* (75%).

A Tabela 2 apresenta a porcentagem de concordância entre os membros do comitê de especialistas para a análise dos aspectos referentes ao formato do teste.

**Tabela 2:** Concordância positiva (%) com relação ao formato geral do teste (n=14)

QUESTÃO	RESPOSTAS				%
	Totalmente adequado	Razoavelmente adequado	Pouco adequado	Inadequado	
Instruções	10	3	1	-	93%
Interface	11	4	-	-	100%
Formato da resposta	10	5	-	-	100%
Escolha das canções	12	2	-	-	100%
Tempo de duração	9	6	-	-	100%

Uma vez concluída a fase quantitativa de validação de conteúdo do BATUTA deu-se início ao processo de análise qualitativa das respostas descritivas oferecidas pelo comitê de especialistas sobre “O que poderia melhorar no BATUTA”. Desse modo, as observações e recomendações dos especialistas foram criteriosamente lidas e analisadas, conforme descrito no Quadro 2, de modo a elencar categorias constituídas a partir de suas falas^(25)^.

**Quadro 2:** Categorização e trechos extraídos das falas dos especialistas com as adequações promovidas

CATEGORIAS	TRECHOS EXTRAÍDOS QUE APONTAM (A) / SUGEREM(S)	ADEQUAÇÕES
Duração das amostras	(A) exemplos longos para o objetivo proposto/duração das gravações estendida	Formatação das frases musicais
	(S) mais tempo de silêncio entre os exemplos	Formatação dos vídeos com ajuste do tempo de apresentação das gravações e silêncios
	(A) diferença de andamento entre as gravações	Regulação do andamento das gravações como software *Audacity* ® 2.3.1.
Qualidade do áudio	(A) ruídos nas amostras	Aplicação do efeito *noise reduction* do software *Audacity* ® 2.3.1
	(A) eco	Aplicação dos recursos *fade in* e *fade out*
	(A) chiados ao fundo	
	(A) corte abrupto dos sons	
Intensidade das amostras	(A) gravações com volume mais forte que as outras	Normalização das gravações com o software *MP3Gain* 1.3.4.
	(A) amostras do teste de timbre com intensidades diferentes	
	(A) diferença entre os ‘volumes’ de algumas gravações	

Assim, a gravação dos estímulos sonoros do subteste harmonia, cuja qualidade foi classificada como *regular* por quatro especialistas e *ruim* por um deles, foi corrigida com novas gravações. No que se refere ao cumprimento desse mesmo subteste, foi possível relacionar os resultados da avaliação dos especialistas à qualidade dos áudios, a partir dos comentários sobre a presença de eco nas amostras, e a regravação corrigiu os problemas apontados para o item harmonia.

As observações dos especialistas referentes às instruções e às consignas no início do teste, ou antes da apresentação das amostras sonoras, resultaram em orientações mais detalhadas ao início de cada módulo e/ou subteste.

### Aplicação do teste piloto

Após as adequações deu-se início à aplicação do teste piloto com os 51 participantes, que ouviram cada uma das 35 amostras sonoras, e responderam *igual/diferente* para as perguntas referentes aos módulos: (1) ritmo: *as amostras são*?; (2) *pitch: os trechos da canção são?*/*os acordes são*?; (3) timbre: *os trechos da canção são tocados por instrumentos?*

Estiveram presentes na sala durante a realização do teste piloto o participante e o examinador^^7^^ . O tempo médio de resposta foi de 20 minutos as repetições foram permitidas, embora não fossem encorajadas.

### Resultados do teste piloto

Os resultados dos 51 participantes, considerando os valores de 1 (um) e 0 (zero), atribuídos para as respostas igual/diferente das 35 amostras sonoras, assim como dos módulos do Batuta, estão descritos na Tabela 3.

**Tabela 3:** Valores de média, desvio padrão, mediana, mínimo e máximo das amostras avaliadas (n=51)

	n	média	desvio padrão	mediana	mínimo	máximo
Ritmo	9	8,4313	0,8307	9	7	9
*Pitch*	16	15,0980	1,2042	16	11	16
Timbre	10	9,8235	0,4338	10	08	10
Total	35	33,3594	3,5355	34	29	35

No que se refere ao desempenho dos participantes no teste, os resultados revelaram que o menor índice foi 82%, correspondente à resposta correta para 29 amostras, e 54,89% dos participantes obtiveram resultados acima da média. O número de acertos, o índice de acertos e a proporção de participantes com valores iguais ou superiores para cada faixa de acertos estão descritos na Tabela 4.

**Tabela 4:** Resultados do teste piloto do BATUTA (n=51)

Número de acertos dos participantes	Índice de acerto dos participantes (%)	Número de participantes que acertaram as respostas	Índice de participantes que acertaram as respostas (%)	Índice de participantes que acertaram um número igual ou maior (%)
29	82%	2	3,92%	100%
30	85%	3	5,88%	96,05%
31	88%	4	7,84%	90,17%
32	91%	5	9,80%	82,33%
33	94%	9	17,64%	72,53%
34	97%	8*	15,68%*	54,89%*
35	100%	20*	39,21%*	39,21%*

* Porcentagem de participantes que apresentaram resultados acima da média

### Validação de consistência interna

O teste de Kuder-Richardson (KR-20) foi utilizado na avaliação da consistência interna do teste piloto. O resultado para as 35 questões com respostas dicotômicas, expressas em igual/diferente, estimado pela análise estatística foi de 0.62.

### Validação de confiabilidade

A confiabilidade do BATUTA foi validada por meio do teste-reteste conduzido com 14 participantes extraídos do grupo inicial. Os resultados das duas aplicações do teste foram utilizados para o cálculo do coeficiente de *Kappa* (K) que resultou no valor de 0,89.

## DISCUSSÃO

Apesar do avanço nos instrumentos de avaliação disponíveis para a prática fonoaudiológica, um número limitado é submetido ao processo de validação em busca de evidências por sua homologação^(18)^. Além disso, há falta de diretrizes na construção e no uso de testes em Fonoaudiologia^(26)^.

No campo da percepção musical, a literatura nacional é representada por um instrumento desenvolvido para avaliar o reconhecimento de melodias tradicionais brasileiras e investigar o desempenho de crianças com audição normal^(27)^.

O BATUTA, nesta mesma tendência, apresenta a singularidade de conter trechos do cancioneiro folclórico brasileiro e é o primeiro teste de percepção musical que avalia os atributos ritmo, *pitch* e timbre, desenvolvido para a população brasileira. Posto que que a música não é um fenômeno culturalmente neutro, é razoável considerar que este é um aspecto promissor do teste.

A realização de uma revisão sistemática sobre os testes de percepção musical em pessoas com deficiência auditiva, previamente à construção do BATUTA, produziu evidências que possibilitaram contornar dificuldades encontradas em estudos anteriores relacionados à heterogeneidade presente na música^(6)^. Ademais, foi possível sistematizar as diretrizes a respeito de elementos musicais avaliados, ambiente do teste, modo de apresentação dos estímulos sonoros e tipo de resposta adequados para a testagem proposta, de modo a estruturar os conceitos e a argumentação da função medida para a elaboração de um construto robusto^(19)^.

Um exemplo deste contexto é o resultado da metanálise que evidenciou a dificuldade maior dos usuários de IC na percepção da melodia do que na percepção do timbre, em comparação aos grupos de ouvintes, com o timbre avaliado a partir de sons digitalizados e os testes de melodia feitos com amostras sintetizadas^(19)^. Ainda que o público pesquisado tenha sido de usuários de IC é possível relacionar estes achados ao tipo de estímulo sonoro aplicado nos testes, pois os filtros e algoritmos que governam a programação de AASIs e ICs fornecem uma faixa dinâmica reduzida e, desse modo, ouvir um som sintetizado por meio de AASI tende a ser mais desafiador. Este resultado motivou a gravação das amostras sonoras dos módulos *pitch* e timbre com instrumentos reais, ao invés da geração de sons sintetizados.

Sendo a validação de conteúdo um fator determinante na escolha e/ou na aplicação do instrumento, a seleção dos especialistas para o cumprimento desta etapa considerou a formação, a qualificação e a disponibilidade dos profissionais, que foram considerados *experts* neste domínio, já que atuaram como juízes e atestaram a clareza, pertinência e fidedignidade do BATUTA^(24)^.

A porcentagem de concordância entre os membros do comitê de especialistas quanto ao formato do teste foi acima de 90% para todas as questões, o que é desejável^(16)^. Visto que não houve nenhuma resposta para *inadequado* pode-se concluir que a interação dos especialistas com o BATUTA foi boa.

A triangulação dos resultados do IVC com as categorias, ou eixos temáticos, propostos a partir das respostas do que poderia melhorar no BATUTA, permitiu a correlação dos dados objetivos com o conteúdo descritivo e garantiu rigor e objetividade à análise dos argumentos expressos pelos especialistas. A partir destes dados foi possível implementar melhorias nas amostras sonoras para alcançar os objetivos propostos.

É interessante mencionar os resultados que indicam a presença de padrões de audição diferentes entre leigos, estudantes e professores de música, sendo os professores aqueles que utilizaram um repertório maior de critérios técnicos para a análise de *performances* ^(28)^. Assim, é possível inferir que os especialistas foram exigentes na avaliação do BATUTA e que o atendimento às recomendações é um indicador de qualidade do teste.

A análise dos resultados do teste piloto demonstrou que 54,89% dos participantes apresentaram resultados acima da média para os 35 itens pesquisados e que mesmo aqueles com menor resultado obtiveram um número satisfatório de acertos. Isto é, os participantes que acertaram 29 questões, tiveram um índice de 82% de acerto.

Diante deste cenário, é possível concluir que as respostas dos participantes foram consistentes. A fundamentação do BATUTA em protocolos desenvolvidos para avaliar a percepção musical de pessoas com DA, e o conjunto de dados homogêneos na aplicação do teste piloto participantes ouvintes, permitem inferir que sua aplicação na testagem da percepção musical de pessoas com DA, usuárias de AASI, é viável.

Convém apontar que as condições de testagem recomendadas para pessoas com DA, usuárias de AASI, são as mesmas descritas na metodologia aplicada aos participantes ouvintes, à exceção da intensidade de apresentação dos estímulos. Tal como evidenciado nos estudos incluídos na revisão sistemática, propõe-se que este público tenha liberdade para ajustar o volume dos estímulos, apresentados em uma sala silenciosa por meio de caixa acústica, a um nível de audibilidade confortável^(19)^.

A confiabilidade do BATUTA, avaliada pela consistência das medidas realizadas nas condições de teste-reteste, verificou a concordância das respostas entre participantes por meio do cálculo do coeficiente *Kappa*, que pode variar de -1 a 1^(29)^. A interpretação do coeficiente sugere o valor de 0,89 para como indicador de ótima concordância e representa a confiabilidade do BATUTA^(24)^.

O coeficiente de *Kappa* foi escolhido por ser recomendado para avaliar medidas de concordância na área da saúde e considerado um índice útil para a validar a concordância em instrumentos com categorias nominais^(16)^.

Convém mencionar que a confiabilidade também é tratada como fidedignidade, equivalência, consistência, objetividade, reprodutibilidade, estabilidade e homogeneidade, a depender da literatura utilizada e do aspecto do teste destacado no estudo^(30)^.

Dentre as opções para análises da consistência interna de um instrumento de medida encontram-se o teste de *Kuder-Richardson* e o coeficiente *alpha de Cronbach*. Apesar do coeficiente de *alfa de Croncach* ser o tipo de teste mais utilizado, a análise de consistência interna por meio da técnica de *Kuder-Richardson* (KR-20) é recomendada para escalas com respostas dicotômicas, como no caso do BATUTA, cujas opções são igual/diferente.

Tanto na interpretação do coeficiente de *alfa de Cronbach* quanto dos valores de *Kuder-Richardson* recomenda-se de que resultados superiores a 0,70 sejam considerados ideais, embora este valor não seja um consenso. Alguns estudos apontam valores próximos a 0,60 como satisfatórios, o que conduz à aprovação da consistência interna do BATUTA com o resultado de 0,62^(17)^.

Finalmente, a partir da fundamentação apresentada, propõe-se como interpretação dos resultados do BATUTA que cada resposta correta pontue 1,0 (um ponto) e que, ao serem somados todos os acertos, os escores finais sejam desta forma analisados: ≥ 33 acertos (acima de 94%): ótima percepção musical; 29 a 32 acertos: boa percepção musical; 25 a 28 acertos: percepção musical razoável; ≤ 24 acertos (abaixo de 68%): dificuldade na percepção musical.

## CONCLUSÃO

Foram apresentados a teoria e o construto por meio dos quais o BATUTA foi desenvolvido, assim como as justificativas para sua concepção e a população alvo a que se destina.

O cumprimento de diretrizes estabelecidas em testes e protocolos destinados à testagem da percepção musical em pessoas com DA, e os resultados das etapas de validação de conteúdo, de consistência interna e de confiabilidade do teste piloto em pessoas com audição normal, indicaram a viabilidade do BATUTA para a avaliação da percepção musical em pessoas com DA, usuárias de AASI.

O BATUTA é passível para aplicação na população a que se destina e futuros estudos poderão ser conduzidos com o objetivo de comparar a percepção musical entre ouvintes e pessoas com deficiência auditiva, entre usuários de AASI com prescrições de regulagens específicas para a apreciação da música, entre usuários de AASI e IC, e tantas outras possibilidades voltadas a pesquisar e a percepção musical desse público.

O BATUTA tem o potencial de fornecer uma perspectiva inovadora no que diz respeito ao atendimento fonoaudiológico, tanto na fase de seleção e indicação do AASI, quanto no acompanhamento dos usuários de dispositivos auditivos auxiliares que desejem ter acesso ao universo musical.

## Footnotes

1 Do italiano *battuta*. Substantivo feminino: Varinha curta com que os maestros regem as orquestras. Adjetivo: Camarada; confiável. Substantivo: Algo excessivamente bom; primoroso.

2 O *Forced choice method,* é um padrão de resposta utilizado para medir experiências subjetivas^(20)^.

3 Palavras em italiano são comumente utilizadas na notação musical para indicar aos intérpretes como a música deve ser tocada, foram usadas de início por compositores italianos no século XVII e espalharam-se por todo o mundo, desde então. Os termos *accelerando* e *ritardando* estão relacionados à mudança de andamento e significam “gradualmente mais depressa” e “abrandar gradualmente”, nesta ordem.

4 Luiz Gonzaga e Humberto Teixeira, 1947.

5 Acordes são grupos de notas tocadas ao mesmo tempo e combinadas de acordo com uma ordem e uma lógica que definem a harmonia da música. A estrutura de um acorde é formada por três notas musicais: a nota fundamental, ou tônica, uma terça e uma quinta. Existem diferentes tipos de terças e de quintas e suas combinações podem gerar acordes variados, tais como Maior, Menor e Diminuto.

6 Canção folclórica brasileira.

7 Os protocolos sanitários e educacionais de prevenção a COVID-19 foram cumpridos integralmente durante a aplicação.
